# Country-Level Bibliometric Analysis of Edible Insect Research: Geographic Distribution and Contributions to Advancing Sustainable Alternatives for Food and Feed

**DOI:** 10.1155/ijfo/8837527

**Published:** 2025-06-27

**Authors:** Budi Wardiman, Asmuddin Natsir, Syahriani Syahrir, Ulva Dianasari, Ardianto

**Affiliations:** ^1^Department of Agricultural Science, Faculty of Postgraduate, Hasanuddin University, Makassar, Indonesia; ^2^Department of Nutrition and Animal Feed, Faculty of Animal Husbandry, Hasanuddin University, Makassar, Indonesia

**Keywords:** alternative protein, bibliometric analysis, country-level analysis, edible insects, emerging themes, functional foods, geographic distribution, sustainable feed

## Abstract

This study examines global research trends in edible insects using a bibliometric approach to evaluate country contributions, which are essential for understanding the geographic distribution of research capacity, funding availability, and regional priorities. Country-specific insights highlight disparities in research output and infrastructure, providing a foundation for exploring how different nations adopt edible insects in food systems and feed applications. Based on 2291 articles indexed in the Scopus database from 2005 to 2024, the analysis utilized Bibliometrix in R software and VOSviewer for bibliometric visualization. To enhance data processing and presentation, Scimago Graphica, Tableau, and MS Excel were employed for advanced visualizations. The findings reveal the rapid growth in edible insect research, with Europe leading in output, particularly from Italy, Belgium, and the Netherlands. Asia shows strong contributions, with South Korea and China emerging as key players supported by robust funding frameworks. The United States, United Kingdom, and the Netherlands host the largest number of journals, facilitating widespread knowledge dissemination. Collaborative networks, led by Germany, Italy, and Kenya, drive advancements, while the Netherlands ranks highest in citations, underscoring the impact of its research. Emerging themes include bioactive compounds, functional foods, circular economy practices, and sustainable feed for livestock and aquaculture, aligning with global sustainability goals. Insects like black soldier fly larvae, crickets, and mealworms are being explored as efficient protein sources for animal feed. Addressing food safety, allergenicity, and cultural barriers remains critical. Future research should focus on scalable farming, innovative food processing, and underutilized species, with global collaboration and sustainability alignment being pivotal.

## 1. Introduction

Food supplies for a continually growing population pose increasing challenges for the global food system. This is largely attributed to agricultural expansion, recognized as a leading driver of widespread deforestation and land degradation worldwide [1, 2]. According to the Food and Agriculture Organization (FAO), nearly 90% of global deforestation is accounted for by agriculture, a figure much higher than previously estimated [3, 4]. These environmental changes not only destroy ecosystems but also lead to soil erosion, reducing agricultural productivity and threatening food security. The United Nations Environment Programme (UNEP) identifies agriculture as the main cause of biodiversity loss. Intensive farming accelerates soil degradation and disrupts water cycles, further diminishing food production capacity [5, 6]. Additionally, significant portions of grasslands, savannas, and temperate and tropical forests have been lost globally [7]. These alarming environmental impacts underscore the urgent need for sustainable resource management to ensure future food security [8, 9].

In response to these challenges, edible insects have gained attention as a sustainable and efficient alternative protein source. They require significantly less land, water, and feed than traditional livestock while emitting fewer greenhouse gases [10, 11]. Containing up to 70% protein by dry weight and rich in essential amino acids, vitamins, and minerals, edible insects are comparable to conventional protein sources like beef and chicken [12–14]. Moreover, they are high in monounsaturated and polyunsaturated fatty acids, contributing to healthier lipid profiles [10, 15].

Insect farming offers substantial advantages over traditional livestock production. For example, crickets require approximately six times less feed than cattle to produce the same amount of protein [16, 17]. This high resource efficiency makes insect farming an excellent solution for meeting the increasing global demand for protein sustainably. Beyond their nutritional benefits, edible insects are also emerging as functional foods due to their bioactive compounds, including antioxidants, peptides, and chitin, which have been linked to improved gut health and immune modulation [18, 19]. This dual potential positions edible insects as both a sustainable protein source and an innovative contributor to functional food development, aligning with global trends toward personalized nutrition and health-oriented diets. Recent research highlights that bioactive peptides derived from edible insects exhibit antihypertensive, antioxidant, and antimicrobial properties, making them suitable for developing functional foods aimed at promoting cardiovascular and immune health [20, 21]. Furthermore, studies have shown that chitin and its derivatives, such as chitosan, extracted from insect exoskeletons, have prebiotic effects that support gut microbiota balance, thereby enhancing digestive health and reducing inflammation [22, 23]. Insects like mealworms and black soldier fly (BSF) larvae are particularly noted for their high levels of polyunsaturated fatty acids, which contribute to improved lipid profiles and cardiovascular health [22, 24].

In addition to their health-promoting properties, insect-derived proteins have demonstrated potential for use in specialized diets, including those for athletes and individuals with specific nutritional needs. Research into the bioavailability of insect proteins shows promising results, with studies indicating efficient digestion and absorption of essential amino acids, making them an excellent choice for formulating high-performance diets [25, 26]. The combination of high nutritional value and functional properties underscores the versatility of edible insects in addressing global health challenges, paving the way for their integration into functional food markets worldwide.

Historically, entomophagy—the consumption of insects—has been an integral part of many cultures worldwide. Ancient societies such as those in China, Greece, and Rome valued insects for their nutritional benefits [27–29]. Today, over 3000 ethnic groups in 130 countries include insects in their diets, representing a significant cultural heritage that could support broader acceptance of edible insects in modern food systems [10, 30].

Insects also hold immense potential as sustainable feed ingredients for livestock. Derived from species like BSF larvae, mealworms, and crickets, insect-based feeds are highly protein-rich and contain essential nutrients, providing a viable alternative to conventional feed ingredients like fishmeal and soybean meal [15]. Incorporating insects into animal feed can significantly lower production costs, which often depend on resource-intensive agriculture and marine harvesting practices. Studies have shown that feeding insect-based diets to livestock enhances growth performance and improves health and productivity. For example, broiler chickens fed insect protein exhibit comparable or superior weight gain and feed conversion ratios to those fed traditional diets [31–34]. BSF larva meal has been shown to have immunostimulatory effects due to the presence of chitin, promoting better gut health and reducing pathogenic load in poultry [35–37]. In pigs, replacing soybean meal with insect protein improves growth performance and nutrient digestibility while reducing the ecological footprint of feed production [38, 39]. Additionally, BSF oil, a byproduct of insect farming, is rich in lauric acid and exhibits antimicrobial properties that further enhance livestock health [40].

Similarly, in aquaculture, fish fed insect-based diets demonstrate improved growth rates and reduced disease susceptibility [41, 42]. In aquaculture, insect-based feeds are increasingly used to replace traditional fishmeal due to their cost efficiency and lower environmental impact. Studies have demonstrated that insect meals can replace up to 25%–30% of fishmeal in the diets of farmed fishes without adverse effects on growth performance, nutrient retention, or product quality [43]. Furthermore, the inclusion of insect-derived fats in aquafeeds improves the lipid profile of fish fillets, increasing omega-3 fatty acid levels and enhancing market value [44].

The adaptability of insect-based feed has made it suitable for diverse livestock industries. Initially used for ornamental fish, it has since been adopted for commercial poultry and aquaculture feeds. More recently, research has expanded to include ruminant diets such as those for cattle, sheep, and goats, with promising results in nutrient uptake and methane emission reduction—a critical concern for environmental sustainability in livestock farming [45].

Environmentally, insect-based feed aligns with circular economy principles, as many insect species can be reared on organic waste, converting low-value byproducts into high-quality feed [46, 47]. Insect farming, involving species such as the BSF (*Hermetia illucens*), mealworms (*Tenebrio molitor*), and house crickets (*Acheta domesticus*), has emerged as a sustainable approach to managing organic waste, particularly in densely populated urban areas. These insects efficiently convert organic waste into high-quality protein and organic fertilizer, significantly reducing landfill waste and associated pollution [46, 48]. BSF larvae are particularly valued for their ability to process diverse waste streams, including food scraps, agricultural residues, and municipal waste, while producing biomass suitable for animal feed and soil amendments [49]. Similarly, mealworms and house crickets exhibit high feed conversion efficiency and adaptability, making them suitable candidates for waste valorization in urban settings [50].

Recent studies emphasize the scalability and economic feasibility of integrating multiple insect species into urban waste management systems. These systems not only address the challenges of waste disposal but also generate valuable byproducts, such as protein-rich feed for livestock and aquaculture, as well as biofertilizers that enhance soil health [51]. Urban insect farming has demonstrated the potential to reduce municipal organic waste by up to 50% while creating employment opportunities and fostering environmental sustainability in cities [52, 53].

Government involvement is essential to advancing research on edible insects. Governments can promote progress by fostering collaborations, supporting education, and investing in infrastructure such as research libraries, databases, and laboratories [54, 55]. Training programs and knowledge exchange initiatives can improve research quality and facilitate collaboration between academic institutions, industries, and research organizations [56, 57]. By supporting multidisciplinary training programs, governments can bridge the skills gap and ensure the availability of experts in entomology, food technology, and environmental science [58]. Collaborative platforms, such as public–private partnerships and international research consortia, further enhance innovation by integrating diverse perspectives and sharing resources [59, 60].

Moreover, clear regulatory frameworks are essential to building consumer trust and ensuring the safety of edible insect products. Governments can establish comprehensive guidelines for insect farming, processing, and marketing, which align with global food safety standards [61, 62]. Focused funding mechanisms are necessary to drive long-term innovation by supporting pilot projects, scaling up research, and enabling commercialization of insect-based products [63, 64]. Additionally, governments can promote awareness campaigns to educate the public about the environmental and nutritional benefits of edible insects, fostering wider acceptance in nontraditional markets [63].

Country-level studies that highlight successful government-led initiatives can identify regional strengths, challenges, and opportunities. These studies provide a nuanced understanding of how nations contribute to the development of edible insect research and industry, offering valuable insights into the drivers of innovation and the barriers that must be overcome. By focusing on the unique cultural, economic, and environmental contexts of different regions, country-level analyses enable the formulation of targeted strategies to address food security challenges both locally and globally [64]. Country-level bibliometric analyses also facilitate international collaboration by identifying key contributors, funding agencies, and collaborative networks. These insights can help policymakers and stakeholders design programs that enhance global knowledge exchange and resource sharing. Furthermore, examining the role of governments in fostering edible insect research highlights best practices, such as public–private partnerships, targeted subsidies, and education campaigns, which can be replicated or adapted to other regions [65].

This study is aimed at conducting a comprehensive geographic analysis of edible insect research, addressing the following key research questions:

1. Which countries and continent lead in total research output on edible insects, and how has this changed over time?
2. Which countries host the largest number of journals contributing to edible insect research globally, and how does this impact the dissemination of knowledge in this field?
3. Which countries are the primary origins of authors producing the most research on edible insects?
4. Which countries have the highest number of funding agencies supporting research projects in edible insects?
5. Which countries show the strongest collaborative networks and the most cited countries in this field, and how do they impact research productivity?
6. What are the key themes and emerging topics in edible insect research, as revealed through keyword analysis and citation trends?

To achieve the objectives of this study, a bibliometric method has been employed. Bibliometric analysis offers several advantages in research evaluation, allowing for a detailed examination of publication trends, citation impact, and collaboration patterns. This approach provides a quantitative basis for assessing research productivity and influence [66]. It enables the analysis of various aspects of scientific output, including the identification of key authors, leading institutions, and dominant research topics [67]. Bibliometric tools such as cocitation analysis, keyword mapping, and network visualization facilitate the exploration of thematic structures and emerging trends within a field [68].

The use of bibliometric analysis has grown substantially in recent years, driven by advancements in data analytics and access to comprehensive bibliographic databases such as Scopus and Web of Science [69]. It is now widely applied across disciplines, ranging from social sciences to life sciences, reflecting its versatility and widespread acceptance within the global research community [70]. The application of software tools like VOSviewer [68] and Bibliometrix [67] has further streamlined the process of bibliometric evaluation. Studies such as [71] underscore its role in measuring the societal impact of research, while others emphasize its importance in understanding interdisciplinary collaborations [72].

Research on edible insects using bibliometric analysis has been demonstrated in numerous studies, including [73–76] and [77]. However, based on our review, there is a noticeable gap in bibliometric studies that focus on research distribution using a state-level approach. A state-level bibliometric analysis would not only complement existing studies but also provide actionable insights for policymakers and stakeholders aiming to address regional disparities and enhance research productivity.

## 2. Methodology

### 2.1. Data Source Selection

The dataset for this study was sourced from the Scopus database, which is one of the most relevant sources of peer-reviewed literature. This database was selected because it has an extensive reach of quality works in multidisciplinary areas, for instance, in agriculture, food science, and sustainability [78, 79]. Its indexing features are powerful, and the functionality of searching is impressive and contributes to the accessibility of the relevant publications [69]. In addition, such comprehensive coverage was achieved in part due to the extensive scope of metadata offered by Scopus, which includes citation metrics, affiliation data, and even funding data, all of which are crucial when undertaking a bibliometric analysis [80].

### 2.2. Search Strategy

**Figure 1:**
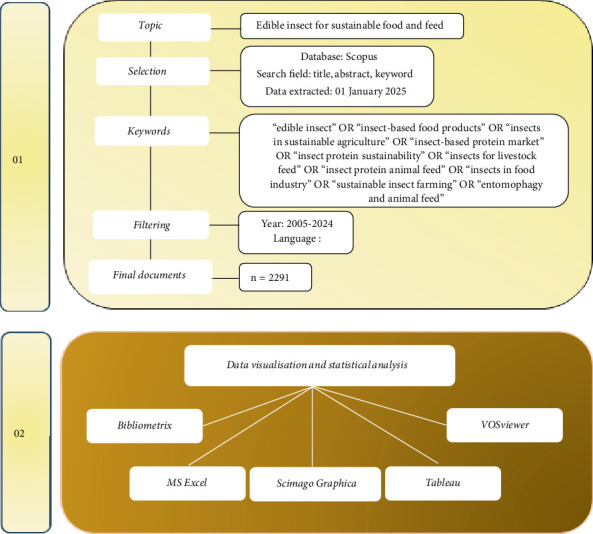
Bibliometric method.

To locate relevant articles around edible insects, a query was developed consisting of keywords which represented the main concept and scope of the study as illustrated by Figure 1. The query included words like title-abs-key (“insect-based food products” or “insects in sustainable agriculture” or “insect-based protein market” or “insect protein sustainability” or “insects for livestock feed” or “insect protein animal feed” or “insects in food industry” or “sustainable insect farming” or “entomophagy and animal feed” or “edible insect”). This limited the sources to publications in English so as to promote reliability and uniformity within the data. Additionally, the timeframe was set between 2005 and 2024 to encompass two decades of research. After applying these criteria, the search yielded 2291 articles focused on edible insects.

### 2.3. Data Processing

The dataset retrieved was exported in CSV format and imported into the R software environment for bibliometric analysis using the Bibliometrix package by [67]. Bibliometrix is an open-source tool created for comprehensive bibliometric analysis and visualization. It allows cleaning and preprocessing of data and provides detailed descriptive statistics and network analyses. Quality assurance for the dataset was done by eliminating duplicate entries and incomplete records, while standardization of authors' names and keywords was performed.

Descriptive insights into the research landscape were provided by key metrics such as the total number of publications, annual growth rate, and average citations per document. Coauthorship, cocitation, and keyword co-occurrence networks were built in order to study collaborative patterns and thematic structures within the field. Advanced visualizations created included thematic maps, trend analyses, and keyword networks for a more developed insight into the research domain.

### 2.4. Additional Analytical Tools

Other tools were added to Bibliometrix, which included VOSviewer, in order to create advance visualizations and analyses. VOSviewer [68] provided high-resolution network visualizations for coauthorship and keyword co-occurrence maps that efficiently handled big sets of data. Other tools used are Scimago Graphica and Tableau for the geographic representation of research contributions and for the article production by nation visualization. Microsoft Excel supported the organization and summarization of numerical data in tabular formats.

### 2.5. Analytical Dimensions

The following dimensions were analyzed and presented to contextualize the research overview of edible insect studies:

1. Scientific production: Analyzed trends in publication output over time and calculated the annual growth rate.
2. Most relevant countries: Identified the leading countries using publication volume as well as scientific impact.
3. Most prolific authors: Assessed author country, productivity, and its citation impact at an individual level.
4. Sources and affiliations: Investigated leading countries that hosted journals, publishers, and institutional affiliations.
5. Citations: Analyzed the most cited countries
6. Content analysis: Assessed dominant themes and emerging topics by keyword analysis and thematic clustering.

## 3. Results and Discussion

### 3.1. Analysis of Publication

**Figure 2:**
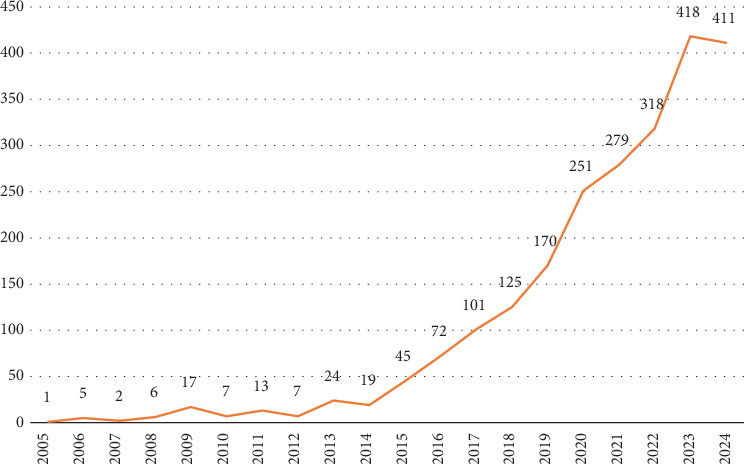
Yearly trend of research publications on edible insects (2005–2024).

The bibliometric analysis of publications related to *edible insects for sustainable food and feed* reveals significant trends over the past two decades (Figure 2), showcasing the growing interest in this field as a solution to global food security and sustainability challenges. By analyzing the distribution of publications from 2005 to 2024, we can observe a significant increase in research output, particularly in the last decade, with a peak in 2023 where 418 publications were recorded. This growth reflects the heightened awareness and research activity surrounding the use of edible insects as an alternative protein source. The slight decline in publications in 2024 suggests a potential stabilization in research output.

Comparing the first decade (2005–2014) with the second decade (2015–2024), there is a striking difference in research output. In the first decade, only about 100 documents were published, whereas the second decade saw a surge with over 2100 publications. This represents a more than 2000% increase in the number of publications, highlighting the escalating interest and importance of this research area. The dramatic rise in publications signifies that numerous aspects remain to be explored and understood. The significant increase in research output points to the vast potential of edible insects as a sustainable food and feed source and underscores the necessity for continued exploration and deeper understanding in this rapidly evolving field.

The annual growth rate of 37.27% highlights the rapid expansion of this research area, supported by a diverse range of topics explored by the 6709 contributing authors (Table 1). The broad distribution of publications across 605 sources suggests a broadening of interest across various academic platforms. The average of 27.63 citations per document demonstrates the impactful nature of this research, emphasizing its relevance in academic and practical discussions about sustainable food systems. With 4900 author keywords and 6969 Keywords Plus, the research encompasses various aspects.

### 3.2. Analysis of Number of Publications by Continent and Country

**Figure 3:**
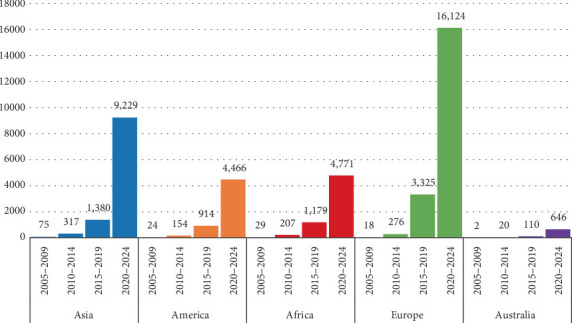
Temporal and regional distribution of edible insect research publications across continents (2005–2024).

Figure 3 demonstrates that Europe leads the global scientific production on edible insects as sustainable food and feed, with a significant margin compared to other continents. Between 2020 and 2024, Europe accounted for 16,124 publications, representing an impressive 45.63% of global contributions. Italy led the region with 5008 publications, followed by Belgium with 1961 and the Netherlands with 1858. The impressive publication output observed across nations is closely tied to the robust funding frameworks established by leading institutions globally. Europe's dominance in research publication is heavily supported by funding from institutions such as the European Commission, which leads globally with 96 funded projects, and the Horizon 2020 Framework Programme, which has supported 41 projects. Belgium, as a major hub of European funding, accounts for 171 funded projects in total, underscoring its central role in driving research on edible insects. These initiatives have positioned edible insects as an emerging solution to reduce the environmental footprint of traditional livestock farming, aligning with the EU's Green Deal and Farm to Fork strategy.

Additionally, Europe's leadership in this area is bolstered by favorable regulations, like the “Novel Foods” legislation (EU No. 2015/2283), providing a clear framework for insect production and marketing, boosting investment and consumer trust [81]. Most European insect production targets the feed sector, with BSF larvae and mealworms dominating, while crickets, locusts, and mealworms are produced for human consumption [82]. Leftover substrates are also repurposed as mineral fertilizer replacements, supporting circular economy practices [83]. Collaborative networks among academia, government, and industry further strengthen Europe's position as a leader in edible insect innovation.

**Figure 4:**
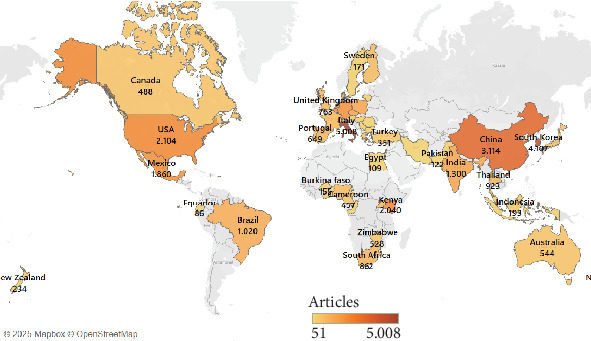
Global distribution of research publications on edible insects by country.

In comparison, Asia emerges as a strong second, contributing 10,998 publications or 25.42% of global publications through 12 countries. Within Asia, South Korea stands out with 4107 publications, followed by China with 3114 and India with 1300 (Table 2 and Figure 4), benefitting from substantial funding as well. South Korea leads with the most research projects in the region, totaling 167 projects across sponsors like the National Research Foundation of Korea (55 projects), Ministry of Science, ICT and Future Planning (39 projects), Ministry of Agriculture, Food and Rural Affairs (37 projects), and Korea Food Research Institute (36 projects). Similarly, China, which ranks third globally, has received 57 funded projects from the National Natural Science Foundation of China (Table 3). These institutions exemplify Asia's growing research capacity and investment in this field. Asia's tropical and subtropical climates naturally support insect farming, enabling diverse species to thrive.

Asia's cultural familiarity with entomophagy continues to be a driving force behind its innovation and leadership in the edible insect industry. Countries such as Korea, India, Japan, and Thailand have long-standing traditions of incorporating insects into their diets. For example, silkworm pupae are popularly consumed in Korea, stinkbugs are a delicacy in India, grasshoppers are often enjoyed in Japan during seasonal festivities, and fried crickets are a staple in Thailand [84–86]. This deep-rooted cultural acceptance has provided a foundation for both traditional and commercial innovation, allowing the region to capitalize on its inherent advantages.

The commercialization of edible insects in Asia is marked by the growth of both small-scale farming and industrial-scale production facilities. Thailand, with approximately 20,000 cricket farmers, has emerged as a leader in cricket farming, catering to both domestic and export markets [87]. Companies such as Bugsolutely in Thailand and Ento in Malaysia are pioneering insect-based products, including cricket flour, burgers, and energy bars. Similarly, Vietnam is home to large-scale production facilities, such as those operated by Entobel, which focus on BSF farming for animal feed and organic waste management [81]. These facilities exemplify how Asia leverages both traditional practices and modern technologies to meet growing market demands.

Institutional and regulatory support also plays a significant role in fostering growth. For example, countries like Thailand and China have implemented safety and quality standards for insect production, particularly for cricket farming, to meet international market requirements such as those set by the European Union [81]. These measures have enabled the export of edible insects and their derivatives, positioning Asia as a critical player in global markets. The development of BSF farms in India and large-scale cricket farms in Korea further demonstrates the region's capacity to expand both its research and commercialization efforts [86, 87]. Asia's leadership in edible insect research is further bolstered by its tropical and subtropical climates, which are ideal for farming diverse insect species [88]. This unique combination of cultural acceptance, favorable environmental conditions, and institutional support has cemented Asia's status as a key player in the global edible insect industry.

Africa, with 14.30% of global publications from 11 contributing countries, also leverages its favorable climate. Kenya (2040 publications) and South Africa (862) lead the continent's output (Table 2). This progress is partly attributed to initiatives like the FAO's 2013 campaign, which promoted insect domestication and led to the establishment of over 2300 small-scale insect farms in sub-Saharan Africa [10, 89]. BSF farming has emerged as a major area of focus, driven by its capacity to convert organic waste into protein-rich feed. Companies such as InsectiPro in Kenya and Maltento in South Africa have advanced BSF production for applications in aquaculture, poultry, and livestock farming [90].

Crickets and grasshoppers also hold considerable significance in Africa. Approximately 26 cricket species are consumed across 25 countries, while grasshoppers like *Ruspolia differens* are widely harvested as a traditional food source [91]. Cricket farming is gaining momentum in countries such as Kenya and Uganda, whereas grasshoppers are still primarily collected from the wild, highlighting the need for more domestication efforts [92, 93].

Financial and collaborative efforts are instrumental in supporting the region's edible insect sector. Noteworthy contributions include Japan International Cooperation Agency's (JICA) $2.5 million investment in Kenya's Regen Organics, the largest BSF facility in the country, and a 3-year BSF project funded by the Norwegian Agency for Development Cooperation in Ghana, Mali, and Niger to improve urban sanitation and promote small-scale farming [89, 94]. These partnerships, combined with Africa's diverse insect biodiversity, provide a strong foundation for the continent's growing role in the edible insect industry. While challenges such as limited financial resources and regulatory frameworks persist, Africa's expanding international collaborations and innovative initiatives indicate promising future growth in both food and feed applications [89, 94].

America, with only five countries contributing, accounts for 12.85% of global publications, led by the United States (2104) and Mexico (1860) (Table 2). Although the region lacks a cultural tradition of insect consumption, increasing awareness of sustainability and the demand for alternative protein sources have driven advancements. America's diverse climates, from tropical zones in Latin America to temperate regions in North America, offer favorable conditions for insect farming. These factors, combined with substantial investments, have helped position the region as an emerging player in the edible insect industry.

In the United States, cricket farming has gained prominence, with at least 11 farms offering products for both food and feed [81]. Companies such as All Things Bugs, which received $5 million in research funding [95], and Chapul Farms, with $2.5 million in financial support [96], have been pivotal in advancing production. Other firms, including Top Hat Cricket and Fluker Farms, focus on mealworms, crickets, and BSFs. Investments in mealworm farming have also scaled up, with Beta Hatch raising $10 million to open a 50,000-square-foot facility in Washington [97]. Additionally, the French company Ÿnsect has expanded into the United States with a Nebraska facility supported by over $500 million in funding [98].

In Latin America, Mexico leads the region in edible insect production, leveraging its biodiversity to farm species like crickets, mealworms, and grasshoppers (*Sphenarium purpurascens*), commonly used in local dishes such as *chapulines*. Brazil and Colombia are beginning to explore insect farming, focusing primarily on animal feed for aquaculture and poultry. Despite this progress, regulatory challenges remain a significant barrier, as many countries lack clear frameworks governing insect farming, processing, and commercialization [29, 81].

Collaboration with international partners has been crucial in addressing these challenges. In North America, the US Department of Agriculture (USDA) has supported projects exploring BSF farming for organic waste management and sustainable feed. Similarly, Latin American countries have partnered with European institutions to improve insect farming technologies. These collaborations are instrumental in establishing safety standards, scaling production, and enhancing export potential [81].

In America, the focus has predominantly been on using insects for animal feed, with BSF and mealworms leading applications in aquaculture, poultry, and livestock. However, interest in edible insects for human consumption is growing. Startups are introducing innovative products like cricket-based protein bars, powders, and snacks. Meanwhile, in Latin America, insects remain primarily used as traditional food, though their application in feed is steadily increasing [81].

Australia and New Zealand contribute only 1.80% of global publications on edible insects (Table 2), but Australia's arid and semiarid climates provide ideal conditions for water-efficient farming of species such as crickets (*Acheta domesticus*), mealworms (*Tenebrio molitor*), and BSFs (*Hermetia illucens*). Over the past decades, these conditions, coupled with growing sustainability concerns, have spurred interest in edible insect farming. However, the sector faces challenges, including limited funding, high substrate costs, unclear regulations, and low consumer acceptance. Most edible insect businesses in Australia are small startups, which struggle to scale production and meet increasing demand [99]. Crickets and mealworms are primarily used for human consumption, often processed into powders and snacks, while BSFs are mainly reared for animal feed and organic waste management. Companies such as Circle Harvest and GoTerra have made strides in producing innovative products, with GoTerra also utilizing BSFs for waste conversion into high-protein feed. These efforts align with sustainability goals and highlight the sector's potential [81]. While funding remains limited compared to regions like Europe, collaborations with international organizations have provided valuable support. Australian institutions, such as CSIRO, have partnered with global leaders like Thailand and the Netherlands to adopt best practices. However, the absence of comprehensive regulations continues to hinder market access and export potential. Public education campaigns and innovative product offerings are vital to overcoming consumer hesitancy and food neophobia [10, 100].

The sharp increase in global scientific output between 2020 and 2024 reflects heightened awareness of edible insects as a viable solution to food security and sustainability challenges. This surge can be attributed to the impact of the COVID-19 pandemic, which exposed vulnerabilities in global food systems, alongside advancements in food technology and increasing funding opportunities [101]. Europe's institutional and financial strength has enabled it to remain at the forefront of this research, while Asia and Africa's contributions emphasize the importance of cultural and natural factors. The Americas and Australia, despite smaller contributions, highlight the role of innovation and policy support in driving research in less traditional regions.

**Figure 5:**
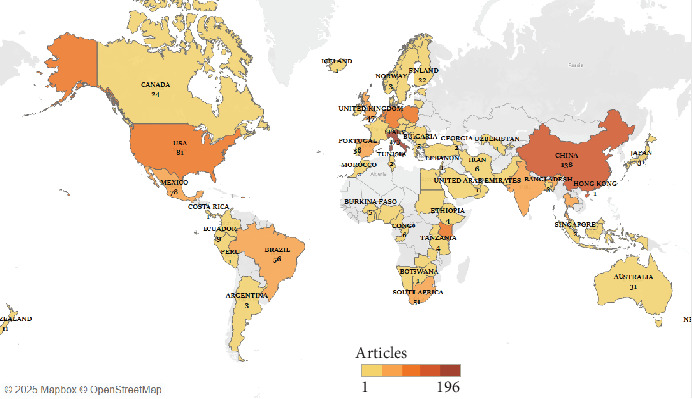
Global distribution of scientific articles on edible insect research based on country of corresponding authors.

The distribution of scientific articles across various countries based on the number of contributions by corresponding authors is illustrated in Figure 5. Korea emerges as the leading contributor with 196 articles. Italy follows with 173 articles and China with 138 articles. These three countries exemplify their prominent roles in driving research and development globally.

### 3.3. Analysis of Article Sources by Country

Table 4 identifies the top 10 significant journals contributing to this field. Among them, the *Journal of Insects as Food and Feed*, published by Wageningen Academic Publishers in the Netherlands, emerges as the most prolific, with 306 articles. This highlights its specialized focus and the growing interest in edible insects for sustainable food systems. Other major contributors include MDPI's *Foods and Insects*, based in Switzerland, which have published 142 and 51 articles, respectively. These journals benefit from MDPI's open-access model, enabling widespread dissemination of research findings. Elsevier's prominent journals, such as *Food Chemistry*, *Food Research International*, and *LWT*, also demonstrate strong representation from the Netherlands, collectively contributing over 100 articles. This underscores Elsevier's pivotal role in advancing food science and sustainability research. Additionally, journals like *Sustainability* (Switzerland) reflect the interdisciplinary nature of the field, bridging sustainability, environmental science, and food innovation.

Table 5 highlights the top 10 countries hosting journals that publish articles in this field. The United States leads with 115 journals, representing 19% of the total, reflecting its robust research infrastructure and significant investments in sustainable food systems. The United Kingdom, with 105 journals (17%), demonstrates its strong focus on food innovation and environmental sustainability. The Netherlands, hosting 98 journals (16%), emerges as a critical hub for food science research, bolstered by key publishers like Elsevier and Wageningen Academic Publishers. Switzerland, home to MDPI and other major publishers, contributes 69 journals (11%), emphasizing its support for open-access research and interdisciplinary studies. Other countries, including Germany (37 journals), Italy (28 journals), India (25 journals), France (19 journals), Canada, and Australia (12 journals each), reflect diverse research perspectives and growing global interest in edible insects.

**Figure 6:**
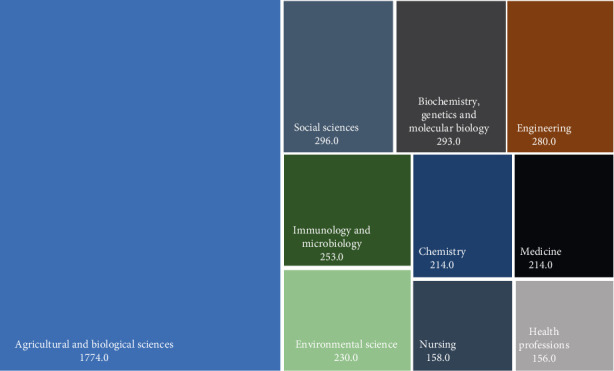
Disciplinary distribution of edible insect research publications by subject area.

The graphical representation of subject areas further underscores the interdisciplinary nature of research on edible insects (Figure 6). The largest share, agricultural and biological sciences (40%), highlights the foundational role of agriculture and biology in understanding insect farming, nutritional value, and sustainable practices. This aligns with efforts to position insects as a viable alternative protein source. Social sciences (7%) emphasize the importance of cultural acceptance, consumer behavior, and policy frameworks in promoting edible insects. Similarly, biochemistry, genetics, and molecular biology (7%) reflect the exploration of nutritional and functional properties of insect proteins, crucial for addressing malnutrition and improving food security. Contributions from engineering (6%) and environmental science (5%) demonstrate technological advancements in processing methods and the ecological benefits of insects, including reduced carbon footprints and efficient resource use.

Additionally, subject areas like medicine (5%), health professions (3%), and nursing (4%) highlight the growing interest in the health implications of insect consumption, such as improved diet quality and addressing nutritional deficiencies. The inclusion of chemical engineering (2%) further underscores the focus on optimizing production processes for scalability. This interdisciplinary scope reflects the potential of edible insects to address multiple global challenges, from reducing greenhouse gas emissions and land use in traditional agriculture to providing high-quality nutrition in food-insecure regions.

### 3.4. Country Analysis by Author

**Figure 7:**
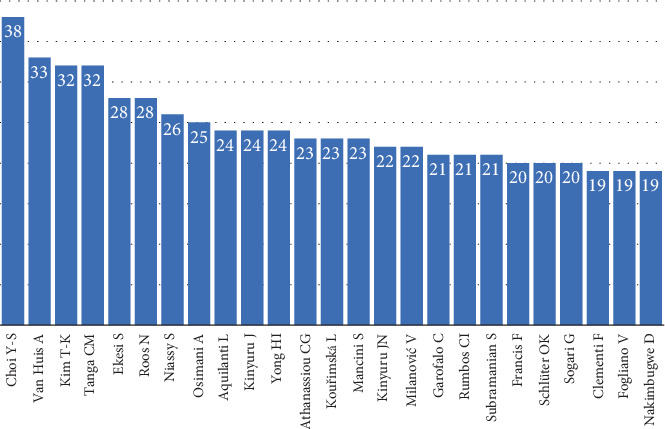
The 20 most prolific authors on edible insect area (2005–2024).

The analysis of author contributions highlights the influential role of leading researchers in advancing edible insect studies worldwide. Yun-Sang Choi from South Korea is at the forefront, with 38 publications, closely followed by Arnold Van Huis from the Netherlands, who has authored 33 papers (Table 6 and Figure 7). Choi's research primarily focuses on processing techniques and functional properties of insects as food ingredients, supporting South Korea's rise in technological innovations in edible insect products [102–105]. Meanwhile, Van Huis has been instrumental in raising global awareness about the ecological and nutritional benefits of entomophagy, with his work for the FAO laying a foundational narrative for edible insects in sustainable food systems [10, 17, 94, 106].

In Africa, researchers like Chrysantus M. Tanga, Sunday Ekesi, and Saliou Niassy, affiliated with the International Centre of Insect Physiology and Ecology (icipe), have contributed to practical advancements, particularly in BSF farming. Their studies have promoted BSF as a sustainable option for livestock feed and organic waste management, directly addressing food security challenges in Kenya and other African nations [89, 90, 107]. These efforts have encouraged local farmers to adopt innovative practices, benefiting regional economies and reducing environmental impacts. Additionally, Kenyan researchers have played a pivotal role in integrating edible insects into urban recycling systems, showcasing Africa's growing potential in sustainable agricultural solutions [108].

European researchers, including Nanna Roos from Denmark and Andrea Osimani from Italy, have deepened the field by addressing consumer acceptance and microbial safety issues. Roos's research explores Northern European consumers' perceptions of insect-based foods, highlighting psychological and market barriers to adoption [109]. Osimani's work, focusing on microbial safety, has guided the development of safety regulations crucial for commercializing insect-derived products in Europe [110, 111].

South Korea, with contributions from Tae-Kyung Kim, has emerged as a leader in developing innovative processing techniques and chemical analyses for edible insects. These advancements have bolstered the country's capacity to scale up insect-based food production, making it a global competitor in the field [112, 113].

**Figure 8:**
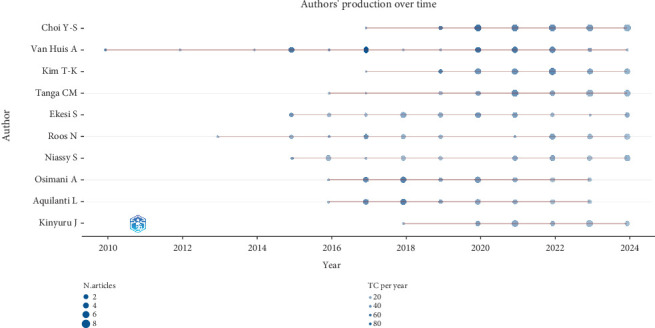
Production over time of top 10 authors on edible insect area (2005–2024).

The consistent outputs of these scholars have shaped global edible insect research, bridging academic insights with practical applications (Figure 8). Kenyan authors' increasing contributions in recent years underscore Africa's growing focus on sustainable solutions. Collaborative networks and funding initiatives have further supported these advancements, emphasizing the interconnected nature of edible insect research in addressing global challenges.

### 3.5. Analysis of Number of Citations by Country

**Figure 9:**
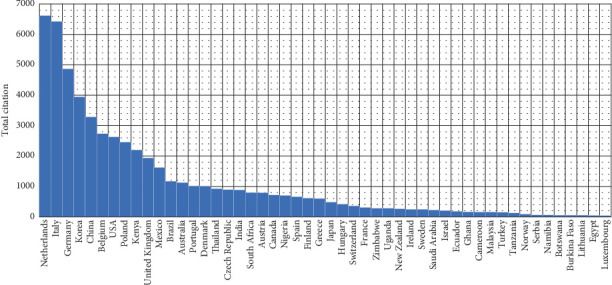
Bar chart of the most cited country on edible insect (2005–2024).

The data highlights the most-cited countries in research on edible insects as sustainable food and feed, showcasing both total citations and average citations per article (Table 7 and Figure 9). The Netherlands stands out as the leading contributor, with 6615 total citations and an exceptional average of 83.7 citations per article, reflecting the high impact and quality of its publications in this field. Italy follows with 6428 total citations but a significantly lower average of 37.2 citations per article, suggesting a greater volume of publications with a more distributed citation pattern. Germany (4870 total citations) and the United Kingdom (1944 total citations) also demonstrate strong contributions, with average citations per article of 46.4 and 41.4, respectively, indicating the notable influence of their research.

Korea (3946 total citations) and China (3287 total citations) rank highly in total citations but have lower averages of 20.1 and 23.8 citations per article, indicating broader but less concentrated citation distribution. Belgium (2733 total citations) and the United States (2628 total citations) maintain a moderate level of contribution, with average article citations of 36 and 32.4, respectively. Poland (2462 total citations) and Kenya (2200 total citations) follow closely, with similar average citations per article of 25.1 and 25.6.

### 3.6. Analysis of Collaboration and Network by Country

**Figure 10:**
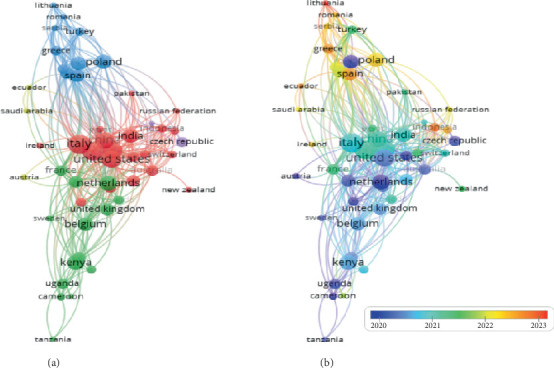
(a, b) Network visualization of collaborative connections among countries in edible insect research.

The collaborative structure in edible insect research, as depicted in Figure 10a, provides an intricate view of global partnerships organized into seven distinct clusters. These clusters reflect varying degrees of regional and international cooperation, underscoring the interconnectedness of research efforts worldwide. It shows seven distinct clusters of countries grouped based on shared coauthorship. Cluster 1 comprises 14 countries: Brazil, Croatia, Greece, Latvia, Lebanon, Lithuania, Mexico, Poland, Portugal, Romania, Serbia, Slovenia, Spain, and Turkey. Cluster 2 includes 13 countries: Austria, Benin, Burkina Faso, Colombia, Côte d'Ivoire, Denmark, Egypt, France, Hungary, Sweden, Thailand, the United Kingdom, and the United States. Cluster 3 features 11 countries: Australia, Ethiopia, Germany, Ghana, India, Indonesia, Malaysia, New Zealand, the Russian Federation, Switzerland, and Taiwan. Cluster 4 consists of 10 countries: China, the Czech Republic, Ecuador, Iran, Israel, Norway, Pakistan, Saudi Arabia, Slovakia, and Sudan. Cluster 5 also includes 10 countries: Argentina, Cameroon, Kenya, Mali, Nigeria, South Africa, Tanzania, Uganda, Zambia, and Zimbabwe. Cluster 6 consists of 9 countries: Bangladesh, Belgium, Canada, the Democratic Republic of Congo, Finland, Japan, Laos, Netherlands, and South Korea. Cluster 7 comprises two countries: Ireland and Italy. These collaborations demonstrate the global interconnectedness of research, with strong regional partnerships in some clusters and broader international networks in others.

Cluster 1, which includes countries such as Brazil, Poland, and Spain, represents a diverse group of nations from Europe and Latin America focused on incorporating edible insects into sustainable agricultural systems. Spain, for instance, has prioritized cricket farming and the extraction of bioactive compounds from insects for functional food products. Collaborative efforts with Italy and Portugal have further strengthened these initiatives, advancing research in food innovation and sustainability [81]. Poland, on the other hand, has concentrated its efforts on sustainable feed solutions, collaborating with research hubs in Germany and the United Kingdom to improve insect farming technologies and develop efficient feed formulations for livestock and aquaculture.

Cluster 2 highlights prominent nations like the United States, the United Kingdom, and France, which have utilized their advanced research infrastructure to lead global collaborations, particularly with Africa and Asia. The United States has invested heavily in developing large-scale farming technologies for mealworms and BSFs. These efforts are complemented by partnerships with European and Latin American countries to facilitate the exchange of knowledge and technologies [10, 12]. Similarly, France has extended its collaboration with Kenya and other African nations, focusing on scaling up BSF farming for waste management and livestock feed production. These partnerships have been supported by international organizations like the FAO, further driving the application of insects as a sustainable solution to global agricultural challenges [114].

Key contributors in the global network include Germany, Italy, and Kenya, which have established extensive connections with 296, 217, and 192 countries, respectively. Germany's prominent position stems from its robust funding ecosystem, supported by initiatives like Horizon 2020 and Horizon Europe, enabling extensive collaborations with European countries such as Belgium and Italy (European Commission, 2022). Italy's expertise in sustainable insect farming has facilitated partnerships with African countries like Kenya and Tanzania, focusing on dual applications for food and feed [115]. Meanwhile, Kenya has emerged as a significant player in Africa, bolstered by partnerships with international donors like the JICA, which invested $2.5 million in Regen Organics, Kenya's largest insect feed industry. These initiatives are aimed at enhancing urban waste management and supporting small-scale farming [116].

Recent trends highlighted in Figure 10b indicate the emergence of countries such as Spain, Poland, Greece, Indonesia, Ecuador, and Lithuania as active contributors to edible insect research. Spain, with its increasing focus on cricket farming and functional food development, has forged stronger collaborations with other Mediterranean and Latin American nations [86]. Poland has intensified partnerships with Germany and the United Kingdom to optimize farming systems and feed production. In Asia, Indonesia has expanded its engagement in insect-based waste management research, collaborating with South Korea, Japan, and Malaysia to develop scalable technologies for BSF farming [81]. Similarly, Ecuador has aligned its research with waste recycling and protein extraction, forming strategic partnerships with European and Latin American institutions to enhance production capacities and technology transfer [117].

Emerging nations such as Lithuania and Greece have utilized European Union funding to focus on niche areas like bioactive compounds and innovative processing techniques, integrating into broader European research networks with countries like Belgium and Italy. African nations, including Cameroon and Uganda, are building their research capacity through collaborations with Germany and France, emphasizing the scaling up of insect farming for animal feed applications.

Efforts to strengthen collaboration are further supported by initiatives that promote global connectivity in edible insect research. European countries continue to allocate substantial resources through programs like Horizon Europe, fostering partnerships with underrepresented research hubs. In Asia, South Korea and Japan have initiated bilateral agreements with countries such as Indonesia and Malaysia, focusing on advancements in insect protein extraction and processing. Additionally, organizations like the FAO and the Norwegian Agency for Development Cooperation have established platforms for knowledge sharing, allowing emerging players like Ecuador and Kenya to adopt best practices in insect farming and waste management [81, 93].

These collaborative efforts underscore the growing recognition of edible insects as a viable solution to address pressing global issues such as food security, sustainability, and climate change. Through international networks, countries are advancing research, fostering innovation, and ensuring the equitable distribution of the benefits associated with edible insect technologies, paving the way for a more resilient and sustainable global food system.

**Figure 11:**
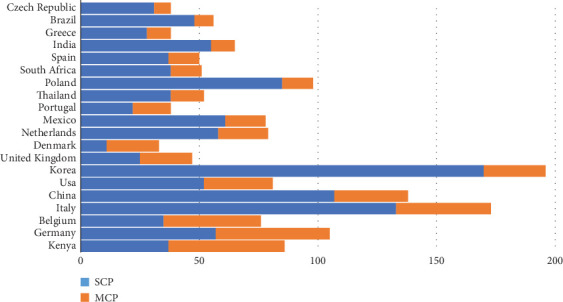
Comparison of single-country and multiple-country publications in edible insect research.

Table 8 and Figure 11 provide a detailed view of research collaboration patterns through single-country production (SCP) and multiple country production (MCP). SCP measures research output conducted solely within a single country, while MCP reflects publications involving international collaboration. According to the data, Korea has the highest SCP (170), followed by Italy (133) and China (107), indicating these countries' robust capacity for independent research. This suggests a strong domestic research infrastructure and focus on self-reliant scientific contributions.

In contrast, countries like Kenya (MCP 49) and Germany (MCP 48) exhibit higher MCP values, indicating significant involvement in international research partnerships. Belgium (SCP 35, MCP 41) also shows a strong balance between independent and collaborative research efforts, suggesting an integrated approach to global knowledge sharing.

The data further highlights the Netherlands with a relatively even split (SCP 58, MCP 21), reflecting its dual strategy of producing impactful domestic research while maintaining consistent participation in international collaborations. Other countries, such as the United Kingdom (SCP 25, MCP 22), demonstrate a more balanced approach, contributing to both single-country and multicountry publications. The balance between SCP and MCP in many countries illustrates the complementary roles of domestic focus and global collaboration in driving scientific progress.

### 3.7. Content Analysis

As shown in Table 9, recent research on edible insects increasingly focuses on their nutritional value, sustainability, and applications as novel foods, reflecting their growing role in addressing global food and feed challenges. The keyword “Edible” dominates with the highest frequency (1184) and total link strength (2384), highlighting its central role in the discourse on edible insects. This aligns with studies emphasizing their potential as sustainable alternatives to conventional protein sources, contributing to food security and environmental preservation.

Keywords such as “Entomophagy” (frequency 415, link strength 1064) and “Novel food” (136, 399) underline the cultural, social, and innovative aspects of consuming insects. These findings align with research by [118] and [119], which explore consumer acceptance and the integration of edible insects into mainstream food systems. The inclusion of “Food safety” (97, 268) and “Sustainability” (97, 292) indicates ongoing efforts to ensure that insect-based products meet safety standards while addressing environmental concerns, consistent with the findings of Baiano [120].

Nutritional aspects are another critical focus, as shown by keywords like “Protein” (87, 290), “Amino acid” (38, 125), and “Fatty acid” (52, 156), reflecting the emphasis on the health benefits of edible insects. These align with studies that investigate the nutritional composition of insects, such as [12], which identified insects as a rich source of essential nutrients. Additionally, keywords like “Mealworm” (73, 173) and “Tenebrio” (108, 270) point to specific insect species frequently studied for their suitability in food and feed applications. These species are explored for their high protein content, easy farming methods, and low environmental impact. This focus is consistent with work published in journals such as the *Journal of Insects as Food and Feed* and *Food Research International*. The presence of terms like “Consumer acceptance” (52, 168) and “Disgust” (24, 67) highlights the social and psychological barriers to adopting edible insects. This reflects efforts to understand and overcome consumer hesitations, as documented by [121]. Furthermore, “Chitin” (31, 92) and “Antioxidant” (34, 90) point to the functional properties of edible insects, suggesting potential applications in health and biotechnology sectors.

We used VOSviewer to create the network visualization, identifying a total of 4871 keywords in the dataset. After filtering for a minimum keyword occurrence of 10, this number was reduced to 116. Subsequently, a thesaurus file was applied to merge similar terms. The use of network visualization is essential for understanding the structure and dynamics of research trends within a field. It enables researchers to identify key themes, prominent keywords, and the relationships between various research topics [68]. These visual tools provide a strategic advantage by helping researchers navigate complex datasets and uncover patterns that might otherwise be difficult to discern. Additionally, visualizing keyword networks aids in identifying influential studies and the collaborative networks driving innovation, allowing policymakers and funding agencies to allocate resources more effectively [70].

**Figure 12:**
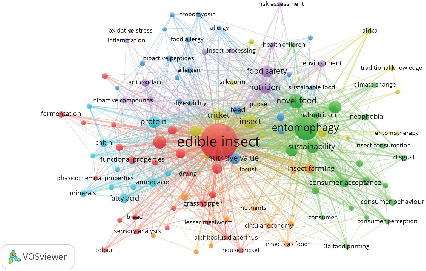
Keyword co-occurrence network visualization in edible insect research.

Figure 12 visualizes the research landscape on edible insects as sustainable food and feed, showcasing various interconnected keywords grouped into clusters, each representing specific thematic focuses in the field. The size of the nodes reflects the frequency of keyword occurrences, with larger nodes indicating greater prominence. Edges between nodes represent co-occurrence relationships, with thicker lines denoting stronger associations. Clusters are color-coded to group thematically related topics.  Table 10 provides additional clarity by identifying key terms associated with each cluster. Cluster 1 emphasizes the biological and nutritional properties of edible insects, with keywords like “protein,” “amino acid,” and “functional properties.” This cluster highlights research exploring the fundamental components that make insects a viable and sustainable source of nutrition.

Cluster 2 focuses on innovative technologies such as “3D food printing” and “novel food.” This area of research explores how modern technology can be applied to make edible insects more appealing and accessible to consumers, particularly in creating new forms of insect-based products. Cluster 3 revolves around allergenic concerns, with terms like “allergy,” “allergenic,” and “risk assessment.” This cluster reflects research addressing consumer safety and the potential risks associated with the consumption of edible insects.

Cluster 4 includes broader environmental and cultural aspects, with keywords like “Africa,” “climate change,” and “traditional knowledge.” This cluster emphasizes the role of edible insects in mitigating environmental challenges and their cultural significance in various regions. Cluster 5 highlights the antioxidant properties of edible insects, as seen in keywords like “antioxidant” and “bioactive compounds.” Research in this cluster explores the potential health benefits of insect-derived components in improving human well-being. Cluster 6 focuses on the physiological and chemical components of insects, with terms like “amino acid,” “fatty acid,” and “chitin.” This cluster investigates the unique biochemical properties that contribute to the nutritional value and functional applications of edible insects. Cluster 7 is centered on insect farming and consumer acceptance, featuring terms like “insect farming,” “sustainability,” and “consumer behavior.” This cluster addresses both the production and market dynamics of edible insects, emphasizing sustainable practices and consumer perceptions.

Through the analysis of keyword clusters and network visualization, it can be concluded that research on edible insects primarily focuses on several key perspectives. From the nutritional perspective, the research emphasizes the potential of edible insects to address global food and feed security challenges and provide a sustainable alternative to traditional protein sources. Studies in this area explore the nutritional benefits of insects and their role in combating malnutrition and meeting dietary needs efficiently.

From the consumer perspective, research delves into understanding consumer behavior, acceptance, and perceptions of edible insects as food. This includes examining factors such as cultural acceptance, willingness to adopt insect-based products, and strategies to overcome barriers like neophobia and societal stigma. These studies are aimed at enhancing consumer trust and increasing the marketability of edible insect products. From the environmental perspective, the focus is on the sustainability of insect farming and its role in mitigating the environmental impact of food production. Research highlights the reduced resource use, climate change, food security, and lower greenhouse gas emissions associated with insect farming, circular economy, and consumer acceptance.

From the technological perspective, the studies address advancements in production, processing, and safety standards for edible insects. The application of modern technologies such as innovative farming systems, food processing techniques, and quality control measures ensures that insect-based products meet safety and regulatory requirements while remaining scalable and efficient.

Despite edible insect research being established over two decades ago, substantial growth in interest and output has only emerged in the past 10 years. The earlier stages of research were characterized by limited findings, with the field still in its embryonic phase, lacking significant focus and clear thematic directions. However, recent years have witnessed a marked increase in attention and collaboration, with stronger linkages forming between research themes, laying a solid foundation for future advancements in the field.

**Figure 13:**
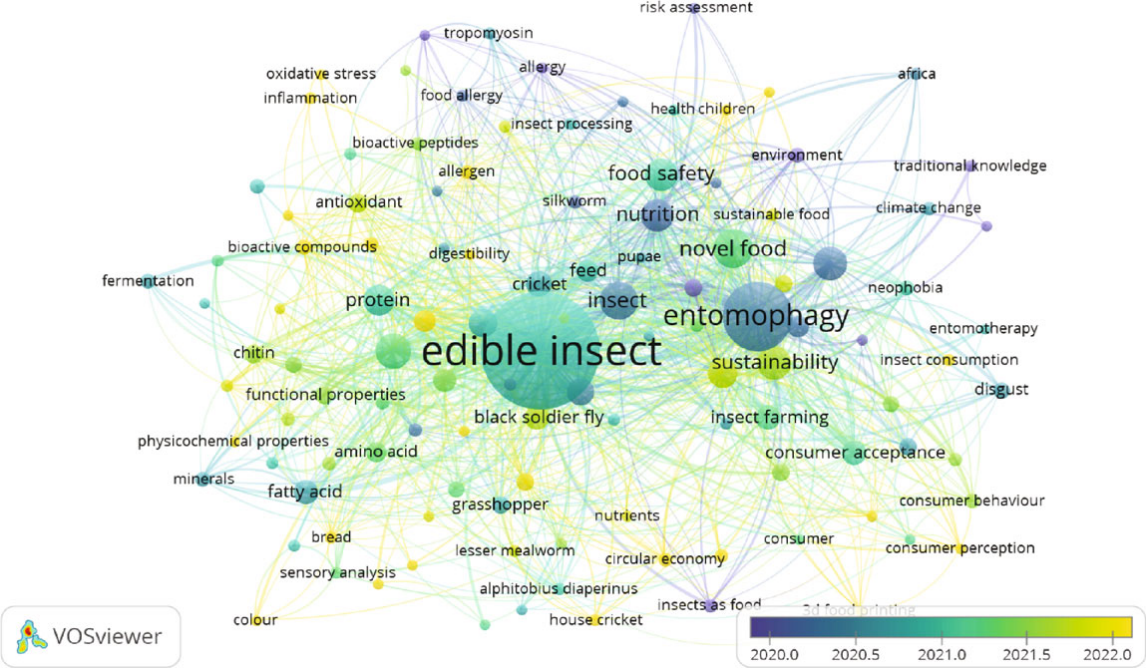
Overlay visualization of edible insect research trends.

Figure 13 is the overlay visualization by VOSviewer showing the evolution of research topics related to edible insects through the mapping of relevant keywords in the field. This overlay is useful for insight into the evolution of research over time and also shows clusters of topics according to their importance over different time periods. The color gradient shows the average year of publication associated with each keyword: darker shades (purple) correspond to older research topics, and lighter shades (yellow) correspond to more recent areas of investigation.

Previous studies, represented by the purple shades, focused primarily on core topics such as “entomophagy,” “nutrition,” and “sustainability.” These are the subjects that underpin initial research into the potential of edible insects as alternative protein sources to meet global food security challenges. Early research also looked at “protein,” “amino acids,” and “functional properties,” representing the nutritional benefits and biochemical characteristics of insects as food. Considerable attention has been given to the ecological benefits of entomophagy, where scholars have assessed their potential to lower greenhouse gas emissions and reduce land use in comparison with conventional livestock production systems [10].

Terminology like “novel food,” “food safety,” “consumer acceptance,” and “insect farming” (in green and yellow shades) highlights present-day research areas. More recent studies have increasingly looked at the challenges associated with integrating edible insects into traditional food systems. These include improving safety protocols in processing, assessing the allergenic potential of such foods (e.g., “tropomyosin” and “allergen”), and studying consumer attitudes in overcoming food neophobia. Contemporary studies underline the importance of sustainable agricultural methodologies, with a particular focus on improving the use of species like “BSF” and “crickets” for food and feed production [32]. Even the concept of a circular economy, coined by terms like “waste reduction” and “sustainable food,” is showing a growing interest in the use of insects in managing organic waste and as a source for livestock and aquaculture.

Contemporary terms such as “bioactive compounds,” “chitin,” “antioxidants,” and “peptides” reflect growing scholarly attention to the potential health benefits of entomophagy or the eating of insects. Studies are under way to determine how these bioactive components may be responsible for improving gut health, enhancing immune response, and reducing inflammation, thus solidifying the use of edible insects as useful components in functional foods and nutraceuticals [25]. Further research is expected to continue to explore food processing innovations, applications of functional foods, and geographical focus expansion in examining underutilized insect species found in biodiversity-rich areas such as Africa and Latin America. Integrating scientific progress with consumer acceptance, the sector of edible insects is poised to change the face of global food systems for the promotion of resilient dietary practices. Another emerging theme pertains to “consumer perception” and “sensory analysis,” through which research efforts have been devoted to finding methodologies that enhance the taste, visual appeal, and commercial viability of insect-derived products to gain consumer acceptance in nontraditional markets.

### 3.8. Future Research Directions

The bibliometric analysis of research related to edible insects reveals several promising avenues for future investigations. These directions do not only try to address the current gaps but also align with global challenges such as food security, sustainability, and nutrition.

#### 3.8.1. Nutritional Improvement and Application in Functional Food

Further research is needed to deeply investigate the bioactive compounds in edible insects, such as chitin, peptides, and antioxidants, and their possible roles in improving gut health, reducing inflammation, and modulating immune systems. The research should be directed to understand how these compounds can be used in the development of functional foods and nutraceuticals, particularly in addressing malnutrition and chronic diseases. More knowledge on nutrient bioavailability and optimization of insect-based diets could further enhance their nutritional appeal.

#### 3.8.2. Scalable Farming and Sustainability

The most important thing would be the development of efficient, sustainable, and scalable agricultural practices related to edible insects. Research should focus on integrating insect farming into circular economic models, utilizing organic waste streams, and reducing environmental footprints. Advanced agricultural technologies, such as automated rearing systems and artificial intelligence–driven monitoring, could help increase productivity while decreasing costs. Research in the life cycle assessment of various insect species can also guide sustainable agricultural strategies.

#### 3.8.3. Consumer Behavior and Market Acceptance

Another major component is to address the psychological and cultural barriers to insect consumption. Future research in this area should focus on the perceptions, preferences, and willingness of consumers to adopt edible insects as food or feed. Cultural attitude insights, particularly for regions where insect consumption is not traditional, could be used to inform marketing and education strategies. Collaboration between social scientists and food technologists can result in products that will meet consumer expectations while overcoming neophobia.

#### 3.8.4. International Cooperation and Regional Specialization

Strengthening global research networks would bring about the needed knowledge sharing and innovation. Cooperation between biodiversity-rich regions of Africa, Asia, and Latin America and high-research-output regions of Europe and North America could help discover new species and uses. Region-specific studies could develop regionally appropriate solutions, allowing insect farming, processing, and consumption to be tailored to meet specific needs.

#### 3.8.5. Processing and Product Technology Innovation

Research on new food processing technologies, including 3D printing and extrusion, can make the products of insects more attractive and approachable. Technological progress in ensuring food safety, reducing the risk of allergens, and improving shelf stability is also of great importance. Innovative formulations, such as insect-based protein powders or snacks, can further diversify product lines and meet global market demand.

#### 3.8.6. Regulatory Frameworks and Policy Support

Development of clear and supportive regulatory frameworks for the production, processing, and trade in edible insects. Research is needed to identify the regulatory gaps, assure the food safety standards, and create policies that encourage investment in the sector. Comparative studies of successful policy models through regions may guide the establishment of globally consistent regulations.

#### 3.8.7. Explore Underutilized Species

Further research is needed on lesser-studied insect species that show promise as food and feed. The studies could be on their nutritional profiles, environmental impact, and economic viability. Moreover, region-specific species need to be looked for to ensure that biodiversity is conserved to meet local dietary needs.

#### 3.8.8. Economic and Social Impact Studies

Further research is needed to examine the economic feasibility of scaling up insect farming and its consequences for the livelihoods of rural people, particularly in developing countries. Research on social impacts of adopting edible insects, including community acceptance and employment generation, can be useful in providing insights to maximize benefits from the industry.

#### 3.8.9. Alignment With Sustainable Development Goals (SDGs)

This will further align studies on insects with international sustainability initiatives, such as the United Nations SDGs, to make them more effective. Investigations should focus on how insect agriculture underpins, for example, Zero Hunger (SDG 2), Responsible Consumption and Production (SDG 12), and Climate Action (SDG 13). Integrating such frameworks into the methodological approaches may increase the societal relevance of investigations related to edible insects.

By following these pathways, scholars, policymakers, and industry players can unlock the full potential of edible insects to achieve sustainable food systems, environmental conservation, and improved nutrition levels globally.

## 4. Conclusion

The findings of this bibliometric analysis highlight significant growth in research on edible insects, with Europe leading in total research output. Countries such as Italy, Belgium, and the Netherlands have emerged as prominent contributors, benefiting from robust institutional frameworks, extensive funding programs, and a strong emphasis on sustainability. This growth has been particularly pronounced over the past decade, reflecting an increasing global awareness of the potential of edible insects as a sustainable protein source. Between 2015 and 2024, research on edible insects expanded exponentially, aligning with global efforts to address food security challenges and reduce the environmental footprint of traditional agriculture. This finding directly addresses RQ1 and underscores Europe's central role in driving research productivity.

In terms of knowledge dissemination, United States, United Kingdom, and Netherlands host the largest number of journals publishing research on edible insects. These countries support key publishers, such as Wageningen Academic Publishers, MDPI, and Elsevier, which have facilitated the widespread distribution of high-quality research findings. The presence of these journals ensures that innovative research reaches a global audience, fostering interdisciplinary collaborations and accelerating the adoption of edible insects as a viable solution to global food and feed challenges. This robust publishing infrastructure addresses RQ2, demonstrating the pivotal role of these nations in shaping the research landscape.

South Korea, Netherlands, and Kenya emerge as leading countries of origin for authors producing the most research on edible insects. South Korean researchers have made significant strides in food processing and technological innovation, while Dutch authors have focused on sustainability and the cultural integration of entomophagy. Kenyan researchers, particularly those affiliated with the icipe, have emphasized practical applications of edible insects to improve food security and nutrition in Africa. These contributions highlight the diverse expertise and regional priorities shaping the field, addressing RQ3 and underscoring the importance of cultivating regional strengths in edible insect research.

Funding plays a critical role in supporting research innovation, and South Korea and China lead globally with 15 funding agencies each dedicated to edible insect research. European nations also play a significant role, with programs like Horizon 2020 providing substantial financial support for research projects addressing sustainability and food security. The availability of targeted funding has enabled researchers to explore novel applications for edible insects, from functional foods to circular economy solutions. This finding addresses RQ4 and illustrates how financial investment drives research productivity and innovation.

Collaboration has emerged as a key driver of progress in edible insect research. Germany, Italy, and Kenya exhibit the strongest collaborative networks, with Germany leading in global research connections with a total link strength of over 290. These networks have facilitated knowledge exchange, integrated diverse perspectives, and enhanced the quality and impact of research outputs. The Netherlands is the most cited country in the field, achieving an average of 83.7 citations per article, which reflects the high quality and relevance of its research. Collaborative initiatives and impactful research outputs address RQ5 and emphasize the importance of fostering international partnerships to advance the field.

The keyword analysis reveals key themes and emerging topics in edible insect research, including sustainability, food security, and nutrition. Emerging areas of focus include bioactive compounds such as antioxidants, peptides, and chitin, which have positioned edible insects as promising functional foods. Circular economy principles, emphasizing waste reduction and resource efficiency, are increasingly integrated into insect farming practices, aligning with global environmental goals. Additionally, technological advancements in farming systems, food processing, and quality assurance are driving innovation in the sector. These findings address RQ6, highlighting the dynamic and multidisciplinary nature of edible insect research.

To build on these findings, governments, research institutions, and industry stakeholders should prioritize strategic investments in interdisciplinary research, establish supportive regulatory frameworks, and implement public education campaigns to enhance consumer acceptance. Promoting international collaborations and leveraging regional biodiversity can further accelerate innovation in edible insect applications. These efforts will ensure that edible insects play a transformative role in addressing global food security and environmental challenges, paving the way for sustainable and resilient food systems.

## Figures and Tables

**Table 1:** General information based on bibliometric data.

**No.**	**Description**	**Results**
1	Timespan	2005:2024
2	Sources (journals, books, etc.)	605
3	Documents	2291
4	Annual growth rate %	37.27
5	Document average age	4.27
6	Average citations per doc	27.63
7	References	102,822
8	Keywords Plus (ID)	6969
9	Author's keywords (DE)	4900
10	Authors	6709

**Table 2:** Regional contribution to edible insect research publications by number of countries, total publications, and percentage (2020–2024).

**Region (number of country)**	**Country (number of publication)**	**Total publication**	**Percentage**
Asia (12 countries)	Korea (4107), China (3114), India (1300), Thailand (923), Japan (493), Turkey (351), Indonesia (193), Israel (166), Malaysia (142), Pakistan (122), Iran (87)	10,998	25.42
Europe (21 countries)	Italy (5008), Belgium (1961), Netherlands (1858), Germany (1831), Czech Republic (1359), Poland (1324), Denmark (992), United Kingdom (763), Spain (749), France (709), Finland (673), Portugal (649), Greece (508), Austria (410), Switzerland (241), Hungary (187), Sweden (171), Serbia (118), Lithuania (92), Romania (89), Bulgaria (51)	19,743	45.63
Africa (11 countries)	Kenya (2040), South Africa (862), Nigeria (716), Uganda (706), Zimbabwe (528), Cameroon (457), Ghana (412), Burkina Faso (155), Congo (140), Egypt (109), Benin (61)	6186	14.30
America (5 countries)	United States (2104), Mexico (1860), Brazil (1020), Canada (488), Ecuador (86)	5558	12.85
Australia (2 countries)	Australia (544) and New Zealand (234)	778	1.80

**Table 3:** Top 10 institutions that provide funding for research on edible insects.

**No.**	**Funding sponsor**	**No. of research**	**Country**	**Continent**
1	European Commission	96	Belgium	Europe
2	National Natural Science Foundation of China	57	China	Asia
3	National Research Foundation of Korea	55	South Korea	Asia
4	Horizon 2020 Framework Programme	41	Belgium	Europe
5	Ministry of Science, ICT and Future Planning	39	South Korea	Asia
6	Brazilian Federal Agency for Support and Evaluation of Graduate Education	38	Brazil	Europe
7	Ministry of Science, Technology and Higher Education	38	Portugal	Europe
8	Ministry of Agriculture, Food and Rural Affairs	37	South Korea	Asia
9	Korea Food Research Institute	36	South Korea	Asia
10	European Regional Development Fund	34	Belgium	Europe

**Table 4:** Top 10 countries that published research on edible insects (2005–2024).

**No.**	**Sources**	**H** ** index**	**G** ** index**	**M** ** index**	**Impact factor**	**Publisher**	**Country**	**Articles**
1	*Journal of Insects as Food and Feed*	35	56	3.182	4.7	Wageningen Academic Publishers	Netherlands	306
2	*Foods*	31	51	4.429	4.7	MDPI	Switzerland	142
3	*Insects*	22	39	2.444	2.7	MDPI	Switzerland	51
4	*Food Chemistry*	24	47	1.412	8.5	Elsevier	Netherlands	48
5	*Food Research International*	26	48	1.625	7	Elsevier	Netherlands	48
6	*International Journal of Tropical Insect Science*	12	22	0.800	1.1	Springer Nature	United Kingdom	40
7	*LWT*	18	33	2.250	6	Elsevier	Netherlands	34
8	*Food Quality and Preference*	20	31	1.818	4.9	Elsevier	Netherlands	31
9	*Sustainability* (Switzerland)	12	18	1.714	3.3	MDPI	Switzerland	31
10	*Frontiers in Nutrition*	13	27	1.857	4	Frontiers Media	Switzerland	29

**Table 5:** Top 10 countries with the highest number of journals publishing articles on edible insects.

**No.**	**Country**	**No. of journal**	**%**
1	United States	115	19
2	United Kingdom	105	17
3	Netherlands	98	16
4	Switzerland	69	11
5	Germany	37	6
6	Italy	28	5
7	India	25	4
8	France	19	3
9	Canada	12	2
10	Australia	12	2

**Table 6:** Top 10 authors who researched edible insects (2005–2024).

**Authors**	**Articles**	**H** ** index**	**G** ** index**	**M** ** index**	**Country**	**Affiliation**
Choi Y.S.	38	17	35	1.889	Korea	Research Group of Food Processing, Korea Food Research Institute, Wanju
Van Huis A.	33	21	33	1.313	Netherlands	Laboratory of Entomology, Wageningen University & Research
Kim T.K.	32	16	32	1.778	Korea	Research Group of Food Processing, Korea Food Research Institute, Wanju
Tanga C.M.	32	13	22	1.300	Kenya	International Centre of Insect Physiology and Ecology (icipe), Nairobi
Ekesi S.	28	16	28	1.455	Kenya	International Centre of Insect Physiology and Ecology (icipe), Nairobi
Roos N.	28	14	28	1.077	Denmark	Faculty of Life Sciences, University of Copenhagen, Copenhagen
Niassy S.	26	13	24	1.182	Kenya	International Centre of Insect Physiology and Ecology (icipe), Nairobi
Osimani A.	25	16	25	1.600	Italy	Department of Agricultural, Food and Environmental Sciences, Polytechnic University of Marche
Aquilanti L.	24	15	24	1.500	Italy	Department of Agricultural, Food and Environmental Sciences, Polytechnic University of Marche
Kinyuru J.	24	9	17	1.125	Kenya	Department of Food Science and Technology, Jomo Kenyatta University of Agriculture and Technology, Nairobi

**Table 7:** The most cited country.

**No.**	**Country**	**T. citation**	**Average article citations**
1	Netherlands	6615	83.70
2	Italy	6428	37.20
3	Germany	4870	46.40
4	Korea	3946	20.10
5	China	3287	23.80
6	Belgium	2733	36.00
7	United States	2628	32.40
8	Poland	2462	25.10
9	Kenya	2200	25.60
10	United Kingdom	1944	41.40

**Table 8:** Top 20 corresponding author's countries.

**No.**	**Country**	**SCP**	**MCP**	**No.**	**Country**	**SCP**	**MCP**
1	Kenya	37	49	11	Mexico	61	17
2	Germany	57	48	12	Portugal	22	16
3	Belgium	35	41	13	Thailand	38	14
4	Italy	133	40	14	Poland	85	13
5	China	107	31	15	South Africa	38	13
6	United States	52	29	16	Spain	37	13
7	Korea	170	26	17	India	55	10
8	United Kingdom	25	22	18	Greece	28	10
9	Denmark	11	22	19	Brazil	48	8
10	Netherlands	58	21	20	Czech Republic	31	7

**Table 9:** The top 20 frequently used keywords on edible insect.

**No.**	**Keyword**	**Freq.**	**Total link strength**
1	Edible insect	1184	2384
2	Entomophagy	415	1064
3	Novel food	136	399
4	Insect	122	281
5	*Tenebrio molitor*	108	270
6	Food security	97	268
7	Sustainability	97	292
8	Nutrition	92	284
9	Food safety	91	252
10	Protein	87	290
11	Alternative protein source	79	237
12	Mealworm	73	173
13	Nutritive value	67	181
14	Cricket	60	181
15	Black soldier fly	53	140
16	Consumer acceptance	52	168
17	Fatty acid	52	156
18	Insect farming	51	145
19	*Acheta domesticus*	50	139
20	Food	41	127

**Table 10:** Keyword clusters.

**Cluster**	**Keywords**
1	*Acheta domesticus*, *Alphitobius diaperinus*, antioxidant activity, black soldier fly, bread, colour, defatting, drying, edible insect, fermentation, functional properties, grasshopper, *Gryllus bimaculatus*, house cricket, insect protein, lesser mealworm, physicochemical properti, protein extraction, sensory analysis, techno-functional properties, *Tenebrio molitor*, *Tenebrio molitor* larvae, texture, volatile compounds, and yellow mealworm
2	3d food printing, acceptablity, alternative protein source, attitude, children, climate change, consumer, consumer accetance, consumer behaviour, consumer perception, cultured meat, disgust, entomophagy, food neophobia, food security, insect consumption, insect food, insect-based food, malnutrition, neophobia, novel food, and sustainability
3	Allergen, allergenicity, allergy, bioactive peptides, digestibility, enzymatic hydrolysis, feed, food allergy, food processing, health benefit, insect meal, legislation, nutritive value, processing, protein hydrolysate, protein quality, safety, silkworm pupae, sustainability food, tropomyosin
4	Africa, biodiversity, cricket, entomotherapy, ethnoentomology, food, insect, larvae, locust, marketing, mealworm, microbiota, pupae, silkworm, and traditional knowledge
5	Antioxidant, bioactive compounds, environment, food safety, functional food, health, inflammation, insect flour, insect processing, nutrition, oxidative stress, risk assesment
6	Amino acid, amino acid profile, chitin, chitosan, fat, fatty acid, fatty acid profile, lipid, minerals, protein, and proximate composition
7	Black soldier fly larvae, circular economy, diet, insect farming, insect as food, insect as food and feed, mass-rearing, nutrients, nutritional profile, *Proteatia brevitarsis*, and protein digestibility

## Data Availability

The original contributions presented in the study are included in the article.
